# Novel *Verrucomicrobiota* strains associated with plant root tissue enhance plant growth and suppress bacterial wilt in tomato

**DOI:** 10.3389/fmicb.2025.1712154

**Published:** 2025-12-17

**Authors:** Minseo Choi, Manigundan Kaari, Hyoung Ju Lee, Seon-Woo Lee

**Affiliations:** 1 Department of Applied Bioscience, Dong-A University, Busan, Republic of Korea; 2 Institute of Agricultural Life Sciences, Dong-A University, Busan, Republic of Korea; 3 Centre for Drug Discovery and Development, Sathyabama Institute of Science and Technology, Chennai, India

**Keywords:** bacterial wilt, enrichment, microbial fraction, plant growth, *Verrucomicrobiota*

## Abstract

**Introduction:**

Rhizosphere microbes interact with plant roots, playing a crucial role in promoting plant growth and alleviating both biotic and abiotic stresses. Our previous study identified putative keystone taxa associated with bacterial wilt (BW) resistance in tomato plants. Among these taxa, bacteria in phylum *Verrucomicrobiota* remain poorly characterized due to challenges in laboratory cultivation. Here, we aimed to isolate novel *Verrucomicrobiota* strains from tomato rhizosphere soil using various carbon and streptomycin-enriched culture media combined with microbiota analysis.

**Methods:**

16S rRNA amplicon sequencing was conducted to identify the suitable carbon source for isolating novel *Verrucomicrobiota* strains from tomato rhizosphere soil. The plant growth promotion and biocontrol assay were conducted to understand the potential of *Verrucomicrobiota* strains. Root hair developmental study was used to observe the tomato root hair formation by safranin-O staining assay.

**Results:**

Microbiota analysis revealed the carbon-source dependent microbial community structure in the enrichment cultures. *Verrucomicrobiota* strains were the most abundant in the cultures enriched with grounded plant roots and streptomycin. A total of 27 novel bacteria, including two *Verrucomicrobiota* strains (V1 and V2), were isolated from different enrichment cultures. *In-planta* plant growth promotion (PGP) assay, strain V2 demonstrated a higher plant fresh weight than strain V1. Disease severity assessment showed that V1 was more effective in controlling BW than V2.

**Conclusion:**

These findings suggest that plant-associated *Verrucomicrobiota* members are involved in plant-beneficial interaction in the rhizosphere. Our study presents a novel strategy for isolating previously uncultivated *Verrucomicrobiota* strains from the tomato rhizosphere using carbon and antibiotic-enriched cultures.

## 1 Introduction

Rhizosphere is a small portion of space that encompasses plant roots and is impacted by plant roots. The rhizosphere is the plant compartment where microbial activity is high. It is also an essential spot for interactions between plants and microbes (Jansson et al., 2023; Compant et al., 2025). These interactions are vital for plant health (Northen et al., 2024) and growth (Trivedi et al., 2020). Microorganisms in the rhizosphere facilitate biogeochemical cycles, the decay of organic matter and the control of soil-borne illnesses of plants, and making them essential for sustainable agriculture (Hartmann and Six, 2023). However, the complexity and diversity of microbial communities in the rhizosphere present significant challenges to harnessing plant-microbe interactions for crop improvement. Previous culture-dependent studies have well-documented the activity of certain bacteria to form beneficial relationships with plants, particularly in the case of well-studied groups like *Pseudomonadota, Actinomycetota* and *Bacteroidota* (Mendes et al., 2013; Trivedi et al., 2020; Pan et al., 2023; Wang et al., 2023; Liu et al., 2024). However, microbes in a rarely represented or not-readily cultivated group seem important in plant-microbe beneficial interaction (Choi et al., 2021). For example, less is known about the contributions of less dominant groups, such as *Verrucomicrobiota*, in plant-microbe interactions. However, they were identified as keystone taxa for plant protection from diseases (Choi et al., 2020).

Bacteria in the phylum *Verrucomicrobiota* are one of the most fascinating yet underexplored groups of soil bacteria. These bacteria are widespread in soil ecosystems and have been identified in several ecosystems, such as agriculture and forest soil (Bergmann et al., 2011; Navarrete et al., 2015). Despite their ubiquitous presence, *Verrucomicrobiota* are often present in low abundance and are notoriously difficult to culture in standard laboratory conditions (Bunger et al., 2020). This has led to a significant gap in our understanding of their ecological roles and potential applications in agriculture. To overcome the challenges associated with isolating novel microorganisms from complex soil environments, various strategies have been developed to modify growth conditions to favor the recovery of slow-growing and fastidious bacteria (Kato et al., 2018; Schultz et al., 2023). Among these strategies, carbon source enrichment and the use of antibiotics have shown promise in selectively enhancing the growth of specific microbial taxa (Wawrik et al., 2005; Wu et al., 2020). The ability of certain bacteria to metabolize complex carbohydrates such as pectin, alginate, and polyols suggests that targeted substrate enrichment could be an effective method for cultivating *Verrucomicrobiota* and expanding our understanding of their metabolic potential (Cha et al., 2021). Meanwhile, antibiotics can reduce the competition by inhibiting the growth of dominant and fast-growing bacteria like *Pseudomonadota*, thereby allowing slow-growing bacteria, such as *Verrucomicrobiota*, to thrive (Amor and Gore, 2022; Schultz et al., 2023).

The diversity of *Verrucomicrobiota* in soil ecosystems and their potential roles in plant-microbe interactions make them an important target to obtain pure cultures and to characterize their activities (Bergmann et al., 2011; Nunes da Rocha et al., 2013). *Verrucomicrobiota* strains are known to be enriched in plant roots as endophytes and contribute to plant growth through various mechanisms (Aguirre-von-Wobeser et al., 2018). It has been suggested that *Verrucomicrobiota* strains may be involved in carbon cycling, the degradation of complex carbohydrates, and processes that are vital for soil fertility and plant growth (He et al., 2017; Aguirre-von-Wobeser et al., 2018; Pold et al., 2018). Moreover, some members of this phylum have been associated with plant roots, where they may contribute to nutrient acquisition and protection against plant pathogens (Bunger et al., 2020). However, the exact mechanisms by which *Verrucomicrobiota* interact with plants and influence soil ecosystems remain largely unknown due to the difficulty in culturing these bacteria.

In this study, we aimed to isolate and characterize novel *Verrucomicrobiota* strains from the rhizosphere of tomato plants. We hypothesized that certain strains of *Verrucomicrobiota* from plant rhizosphere could confer plants with the benefit of promoting plant growth and suppressing plant diseases. This is because our previous study suggested that the *Verrucomicrobiota* members are the keystone taxa in the tomato rhizosphere (Choi et al., 2020). Here our study adopted the use of carbon source enrichment combined with antibiotic selection to isolate *Verrucomicrobiota* strains that have previously been uncultured. This approach expands our understanding of the diversity and function of *Verrucomicrobiota* in the rhizosphere and provides new insights into their potential applications in agriculture.

## 2 Materials and methods

### 2.1 Experimental design

Field soil used in this study was collected from a Dong-A University Agricultural Experiment Station (35° 14’20.4”N 128° 58’40.8”E) located in Daedong-myeon, Gimhae-si, Gyeongsangnam-do, Korea. Microbial fractions (MF) of the field soil were prepared based on our previous study (Choi et al., 2020). In summary, 250 ml of 2.5 mM MES (2-(N-Morpholino) ethanesulfonic acid) buffer (pH 5.7) was added to 170 g of field soil and incubated in rotary shaker at 200 rpm for 30 min. After separating soil particles by centrifugation at 500 rpm for 5 min, the supernatant was centrifuged again at 8,000 rpm for 15 min to get the bacterial pellet. Finally, the MF of the field soil was prepared by resuspending this pellet in 220 ml of 2.5 mM MES buffer (Supplementary Figure 1).

In sterilized petri dishes, surface-sterilized tomato seeds were germinated on sterile filter paper with 5 ml of sterilized distilled water (SDW) for 7 days. Following germination, the seedlings were placed in 45 mm × 45 mm pots filled with 17 g of twice-autoclaved nursery soil (Choi et al., 2020). Following treatment with 20 ml of field soil MF, each seedling was cultivated for 3 weeks at 28 °C with a 14-h light/10-h dark cycle. The plants were taken out from the pots 3 weeks later, and any loose soil was gently shaken off. An ultrasonic cleaner was used to remove soil particles firmly attached to the roots in a 50 ml tube containing 10 ml of 2.5 mM MES buffer. After removing the roots, the rhizosphere soil solution was collected by centrifuging it for 10 min at 13,000 rpm. The soil was then weighed and resuspended in 2.5 mM MES buffer. Further, the suspension was centrifuged again at low speed (500 rpm for 5 min) and then at high speed (13,000 rpm for 20 min) to produce a rhizosphere MF. A rhizosphere MF was extracted by resuspending this pellet in 2.5 mM MES buffer at a density of approximately 10^9^ CFU/ml (Supplementary Figure 1).

### 2.2 Enrichment of carbon source and streptomycin treatment

The primary and secondary enrichment cultures were prepared using the carbon sources listed in Supplementary Table 1. A mineral broth medium was used for both enrichment cultures, composed of the following (g/l): KH_2_PO_4_ (0.5), NaCl (0.2), MgSO_4_ (0.2), NH_4_Cl (0.4), CaCl_2_ (0.1), with a pH range of 6.5–7.0. Individual carbon sources were added to the mineral broth medium at a concentration of 0.5% (w/v). Plant root tissue (PRT), sourced from a 3-week-old tomato, was also used as one of the carbon sources in this experiment. The root tissue (200 mg/plant) was finely ground with sterilized mortar and pestle in sterile distilled water (SDW), followed by centrifugation at 13,000 rpm for 5 min, and the collected pellet was used for enrichment. Then, the collected pellet (100 mg) was mixed with 10 ml of sterile distilled water to makeup as 1% w/v. A total of 52 carbon sources were utilized in primary enrichment cultures, both individually or in combinations with or without antibiotics. These carbon sources were selected based on their reported occurrence in tomato root exudates and rhizosphere metabolites (e.g., sugars, organic acids, amino acids, and polysaccharides), as well as literature indicating their suitability for enriching slow-growing rhizosphere bacteria such as *Verrucomicrobiota* (Dennis et al., 2010; Nunes da Rocha et al., 2013; Ulbrich et al., 2022; Alahmad et al., 2024; Yan et al., 2024). For the secondary enrichment culture, 31 carbon sources were selected based on the primary enrichment results that showed increased *Verrucomicrobiota* abundance and diversity. For the primary enrichment culture, the rhizosphere MF (10 μl/ml) was added to 3 ml of the mineral broth containing 0.5% carbon source, and the mixture was incubated with shaking at 200 rpm at 30 °C. A similar treatment containing streptomycin (25 μg/ml) was added to a similar set of mineral broth under the same conditions, serving as antibiotic-enriched samples. Previous report revealed that antibiotic treatment such as streptomycin significantly alters microbiome and may enrich certain *Verrucomicrobiota* members (Bazett et al., 2016). All the samples were incubated 30 days, depending on the bacterial growth rate observed for each carbon source. The extended incubation period was adopted to allow adequate proliferation of slow-growing taxa such as *Verrucomicrobiota*, which typically exhibit prolonged lag phases and slow cell-division rates in artificial media owing to their oligotrophic lifestyle (Nunes da Rocha et al., 2013; Bunger et al., 2020). Similarly, in the secondary enrichment culture, the rhizosphere MF (10 μl/ml) was inoculated into 5 ml of mineral broth containing 0.5% carbon source selected based on the results from the primary enrichment experiment. A total of 31 carbon sources were utilized for the secondary enrichment culture. The culture was then incubated under the same conditions at 30 °C with shaking of 200 rpm for 2–30 days, depending on the growth rate observed for each carbon source. Later, the microbial cells were obtained by centrifuging 2 ml of the primary and secondary enrichment cultures for 3 min at 13,000 rpm and the microbial pellet was stored in a −80 °C freezer until further analysis. Due to the large number of carbon sources used in the primary enrichment cultures, each condition was tested using a single representative sample. For the secondary enrichment experiments, five biological replicates were performed for each selected carbon source to ensure reproducibility and to support downstream microbiome and sequencing analyses.

### 2.3 Microbial community analysis

Total bacterial DNA was extracted from the enrichment samples stored at −80 °C using the DOKDO-Prep™ Soil DNA Isolation Kit (ELPIS-Biotech, Korea). The DNA concentration was adjusted to 5 ng/μl using a NanoDrop spectrophotometer. Primers 341F (5′-TCGTCGGCAGCGTCAG ATGTGTATAAGAGACAGCCTACGGGNGGCWGCAG-3′) and 805R (5′-GTCTCGTGGGCTCGGAGATGTGTATAAGAGACAG GACTACHVGGGTATCTAATCC-3′) (Mizrahi-Man et al., 2013) were used to amplify the V3–V4 region of the 16S rRNA gene. Primers at a concentration of 1 μM, 2X KAPA HiFi HotStart Ready Mix and 5 ng/μl DNA were used for the PCR amplification. The PCR conditions comprised an initial denaturation at 95 °C for 3 min, followed by 25 cycles of denaturation at 95 °C for 30 s, annealing at 55 °C for 30 s, and extension at 72 °C for 30 s, and a final extension at 72 °C for 5 min. Following purification with Agencourt AMPure XP beads, the amplicons were sequenced at the National Instrumentation Center for Environmental Management (NICEM, Seoul, Korea) using an Illumina MiSeq platform. Microbial community analysis was conducted using the QIIME2 pipeline. Amplicon sequence reads were merged, denoised with DADA2, and clustered into OTUs with 97% sequence identity, with chimeras and singletons removed using VSEARCH. Taxonomic classification was performed using the SILVA database (version 138.2). To investigate the presence of a significant effect of carbon and antibiotic treatments on ß-diversity metrics, the Bray-Curtis dissimilarity matrix was extracted from qiime2 and visualized through the functions of ggplot2, reshape2, and dplyr in R package. Carbon and antibiotic treatments were calculated permutational multivariate ANOVA (PERMANOVA) through pairwiseAdonis2 in R package.

### 2.4 Isolation and identification of microorganisms

The carbon-enriched rhizosphere soil samples were further utilized for bacterial isolation using minimal agar medium containing 0.5% ground root, with or without 25 μg/ml streptomycin, and incubated at 30 °C for 30 days. From the isolation plates, morphologically distinguished bacterial colonies were selected, and sub-cultured in Reasoner’s 2A (R2A) agar and tryptic soy agar (TSA) medium. The bacterial colonies were preserved at −80 °C in 40% glycerol after purification.

A bacterial genomic DNA isolation kit was used to extract genomic DNA from bacterial strains. A 16S rRNA universal primers such as 8F (5′-AGTTTATTGATCCTTAG-3′) and 1492R (5′-GGTTACTTTACGACTT-3′) were used to amplify the bacterial DNA by PCR analysis. A total of 20 μl reaction mixture included 3 μl of DNA, 10 μl of GoTaq^®^ Green Master Mix, 1 μl of each primer (1 μM), and 5 μl of molecular grade water were used. The PCR condition is carried as the initial denaturation at 95 °C for 5 min, followed by 30 cycles of 95 °C for 1 min, 55 °C for 30 s, and 72 °C for 1.5 min, with a final extension at 72 °C for 7 min. The amplified DNA was confirmed by gel electrophoresis and sequenced at Bionics (Seoul, South Korea). Bacterial identification was performed using the EZbioCloud database^1^.

### 2.5 Plant growth promotion of *Verrucomicrobiota* strains

The plant growth promotion assay was conducted in hydroponic and pot culture systems. For the hydroponic method, 4 ml of the bacterial suspension (OD_600_ = 0.2) in sterile distilled water was added to the 5 ml tube. Seven-day old tomato (cultivar Hawaii 7996 and Zuiko) seedlings were placed in the tube containing bacterial suspension, and the surrounding area was sealed to maintain sterility. Sterile water without bacteria was used as a control. The fresh weight of the plants was measured after 9 days of plant growth in a clean room at 28 °C with a 14 h light and 10 h dark cycle. For the pot culture system, 7 days old tomato (cultivar Hawaii 7996 and Zuiko) seedlings were planted in a pot (45 mm × 45 mm) containing 17 g of twice autoclaved (at 121 °C for 40 min) commercial nursery soil (Punong Co., Ltd, Korea). Each seedling was treated with 20 ml of upland MF on the same day and incubated at 28 °C with a 14 h light and 10 h dark cycle. After 3 days, 20 ml of bacterial suspension (OD_600_ = 0.2) was added to each seedling, while the control plants were treated with sterile water. The plants were grown for 35 days in a growth room under the same light and temperature conditions as the hydroponic system. The fresh weight of the plants was measured after 35 days of growth. Both experiments were repeated three times, with 10 plants per treatment group in each experiment.

### 2.6 Root hair developmental study

Fourteen days-old tomato seedlings treated with *Verrucomicrobiota* strains and untreated control samples were observed for root hair formation. Seedlings were stained with 2.5% Safranin-O for 5 min. Then, stained seedlings were washed three times with sterile distilled water and visualized under compound light microscopy.

### 2.7 Biocontrol study

The effect of *Verrucomicrobiota* on controlling tomato BW was investigated by the pot-culture system for both Hawaii 7996 and Zuiko tomato variety. Tomato seedlings were germinated for 7 days in sterile water and planted in nursery soil (17.0 g) in 45 mm × 45 mm pots. After planting, each seedling was treated with 20 ml of *Verrucomicrobiota* V1 or V2 suspension (OD_600_ = 0.2). The plants were grown for 21 days in a growth room with a 14 h light and 10 h dark cycle at 28 °C. For biocontrol efficacy, the pathogenic strains *Ralstonia pseudosolanacearum* SL341 and GMI 1000 were cultured on CPG solid medium amended with 2,3,4-triphenyl tetrazolium chloride (TTC) solution for 2 days at 30 °C. The pathogenic bacterial suspension was prepared using sterile distilled water at 10^8^ CFU/ml (OD_600_ = 0.2). Then, 5 ml of bacterial suspensions containing SL341 or GMI 1000 were poured into pots containing Hawaii 7996 and Zuiko plants, to achieve a final concentration of 10^7^ CFU/g of soil. The plants were monitored for wilting symptoms per individual plant over 14 days under the same growth condition (Kwak et al., 2018).


Wiltincidence(%)=(no.ofwiltedleaves/

totalno.ofleavesofindividualplant)×100

Then, wilt incidence of total plants subjected to disease severity assay was used to generate disease progress curve. The experiment was repeated three times with 10 plants per treatment group in each experiment.

### 2.8 Statistical analysis

Statistical analyses were conducted using R software (version 4.3.2) and QIIME2 (version 2024.2). For community level comparisons, Bray-Curtis dissimilarity matrices were analyzed by permutational multivariate analysis of variance (PERMANOVA) using the pairwiseAdonis2 package in R. In plant growth experiment, significant difference was analyzed by analysis of variance (ANOVA) followed by Tukey’s honestly significant difference (HSD) *post-hoc* test or Kruskal-Wallis rank sum test with Dunn’s *post-hoc* test was performed for pot experiment depending on the data distribution and experimental setup (pot experiments). For hydroponic system, datasets were analyzed by Welch’s ANOVA followed by Games-Howell *post-hoc* test. Biocontrol activity of bacterial strains in tomato against bacterial wilt was estimated for statistical significance by repeated measures ANOVA. All statistical analyses were performed at a significance level of *P* < 0.05, unless otherwise specified. Specific test results and sample sizes are provided in the respective figure legends.

## 3 Results

### 3.1 Carbon-enriched rhizosphere soil microbiota

In total, 671 samples were used in this study to analyze the microbiome. Based on the carbon and streptomycin enrichment, 190 samples were obtained from the primary enrichment culture, and 481 samples were selected from the secondary enrichment culture. A total of 20,559,199 and 53,377,998 high-quality 16S rRNA gene sequences, with a median read count of 107,952 and 114,352 per sample, were obtained from primary and secondary enrichment samples. Likewise, primary and secondary enrichment samples had average OTUs of 1,367 and 1,824, respectively (Supplementary Table 2). The secondary enrichment samples displayed a broad range of diversity, with several samples achieving a high number of observed species, suggesting a rich and varied microbial community.

**FIGURE 1:**
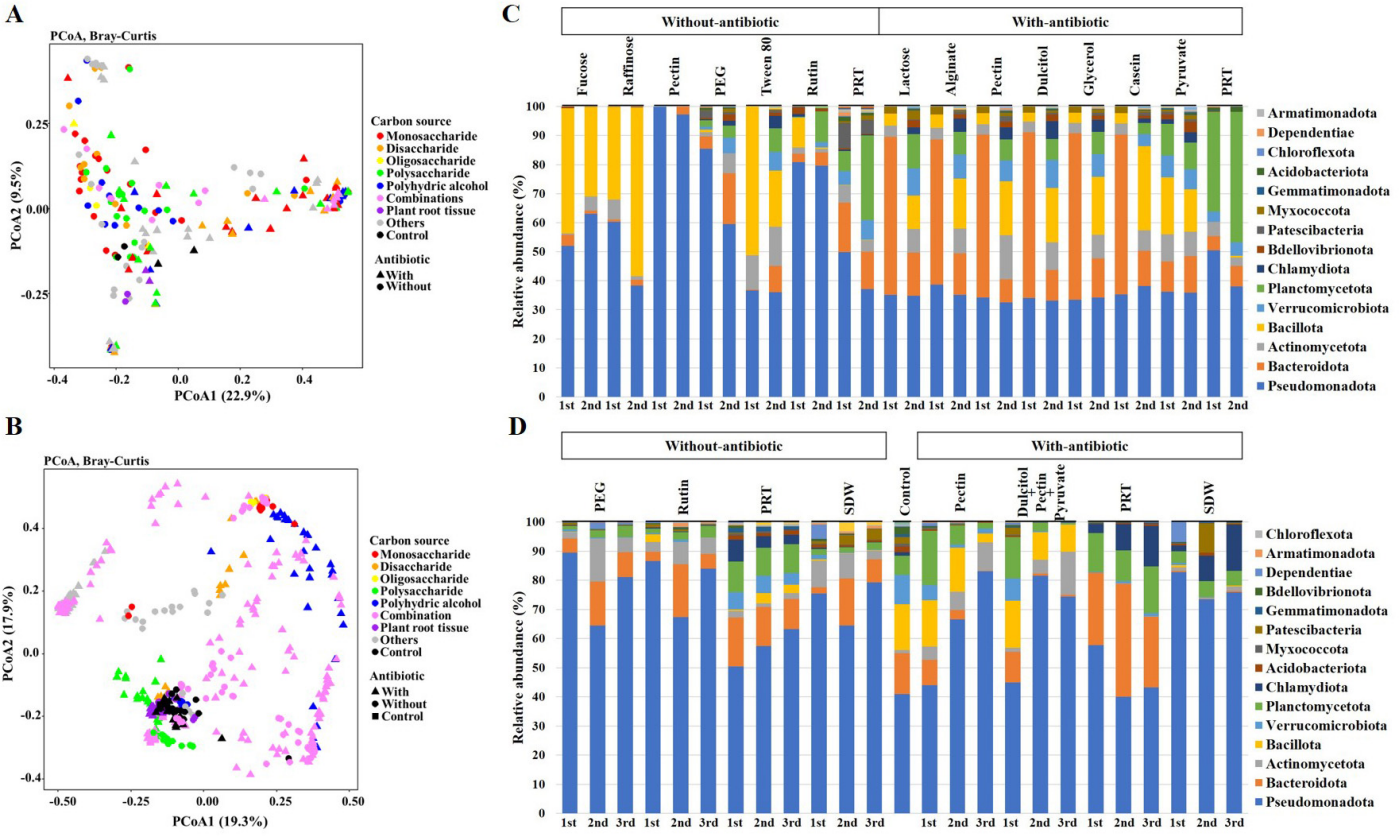
Principal coordinate analysis (PCoA) plot based on Bray-Curtis dissimilarity of bacterial communities from the carbon and antibiotic enriched primary **(A)** and secondary **(B)** cultures. Each symbol indicates microbial community of the enriched rhizosphere microbes cultured in mineral broth enriched with carbon sources alone (▲) or the carbon sources with antibiotic (●). Dot colors shown enrichment by monosaccharides (red), disaccharides (orange), oligosaccharides (yellow), polysaccharides (green), polyhydric alcohol (blue), combination of monosaccharide (pink), plant root tissue (violet), others (gray), and control (black). Microbial community composition of field MF amended tomato rhizosphere soil sample enriched with different carbon sources individually and carbon enriched antibiotics in primary **(C)** and secondary **(D)** enrichment cultures.

The bacterial communities enriched from rhizosphere samples under various carbon sources and antibiotic treatments showed unique clustering patterns according to the principal coordinate analysis (PCoA) based on the Bray-Curtis dissimilarity measure (Figures 1A, B). Across both datasets, distinct clustering patterns were observed based on the type of carbon source utilized. The addition of antibiotics streptomycin also played a crucial role in shaping microbial communities, generally causing a shift in the community structure (Figure 1A). This effect was particularly pronounced in the communities enriched with polysaccharides and plant root tissues, suggesting that these communities were more sensitive to streptomycin treatment (Figure 1B). The statistical analysis revealed that both carbon source and streptomycin treatment significantly influenced bacterial community composition in both primary (Supplementary Figures 2A, B) and secondary enrichment cultures (Supplementary Figures 3A, B), as determined by PERMANOVA (Bray–Curtis dissimilarity; pairwiseAdonis2).

### 3.2 Primary enrichment culture

The microbiome analysis was assessed following the amendment of different carbon sources, individually and in combination with or without streptomycin (Supplementary Table 1). Among 52 carbon sources used in primary enrichment, only 8 and 7 carbon sources were selected and visualized in Figure 1C for conditions with and without antibiotics, respectively. This selection was based on the increased abundance of *Verrucomicrobiota* in sub-cultured samples during primary enrichment. Across all carbon amendments, *Pseudomonadota* remained the dominant phylum (mean relative abundance = 62.58 ± 6.0%), particularly in pectin, PEG, and rutin enriched samples without antibiotics (Figure 1C). In antibiotic-treated cultures, *Bacteroidota* initially increased to 43.15 ± 7.8% but declined to 11.63 ± 1.0% after the second subculture, accompanied by a proportional rise in *Actinomycetota* (8.50 ± 1.1%), *Bacillota* (16.23 ± 2.8%), and *Verrucomicrobiota* (7.25 ± 0.7%). These shifts indicate that antibiotic pressure selectively reduced sensitive *Bacteroidota* members, allowing slower-growing or resistant taxa to proliferate.

### 3.3 Secondary enrichment culture

In secondary enrichments, *Pseudomonadota* accounted for 71.92 ± 3.5% of sequences in most treatments, while *Verrucomicrobiota* increased up to 5.32 ± 0.65% in plant root-tissue amended samples (Figure 1D). The presence of streptomycin decreased *Pseudomonadota* abundance to 63.97 ± 4.9% and enhanced representation of *Planctomycetota* (8.25 ± 1.7%). Collectively, these quantitative results demonstrate antibiotic-mediated restructuring of microbial diversity and the selective enrichment of *Verrucomicrobiota* in carbon-rich environments.

### 3.4 Comparison between primary and secondary enrichments

While the primary enrichment phase captured a diverse assemblage dominated by fast-growing copiotrophic taxa such as *Pseudomonadota* and *Bacteroidota*, the secondary enrichment phase reflected a more specialized and stable microbial consortium. This transition represents an adaptive succession under selective pressure, where prolonged exposure to carbon-enriched and antibiotic-amended conditions favored the persistence of metabolically adaptable or resistant taxa, including *Actinomycetota*, *Planctomycetota*, and particularly *Verrucomicrobiota*. The marked increase in *Verrucomicrobiota* relative abundance suggests that this group successfully adapted to nutrient-rich yet competitive microenvironments. Thus, the secondary enrichment phase likely reflects a stabilized microbial community adapted to sustained carbon and antibiotic selection pressures.

### 3.5 Isolation of culturable bacteria

**FIGURE 2:**
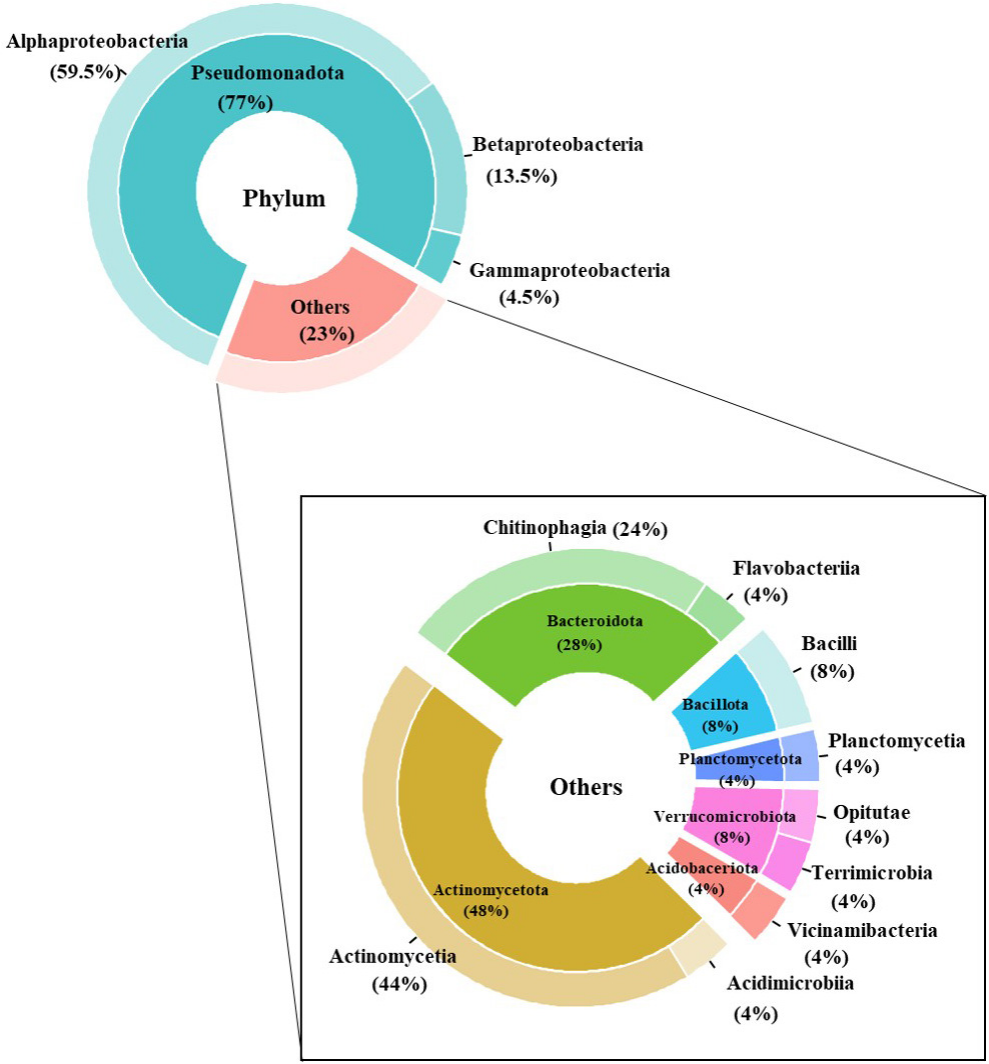
Pie-chart showing the percentage of isolated bacteria. A total of 405 bacterial strains were isolated and indicated at phylum and class level.

A total of 405 strains were isolated and identified at the genus level (Supplementary Table 3). The majority of these isolates belonged to the *Alphaproteobacteria* (59.5%), followed by *Betaproteobacteria* (13.5%) and *Gammaproteobacteria* (4.5%). The remaining strains were distributed among the other (23%) phylum, including *Actinomycetota*, *Bacteroidota*, *Verrucomicrobiota*, *Planctomycetota*, *Firmicutes*, and *Acidobacteriota* (Figure 2). To further explore the diversity within these isolates, 92 unique bacterial strains were selected for their more detailed classification. Notably, 78% of these unique strains were identified as *Pseudomonadota*, followed by *Bacteroidota* (7%), *Actinomycetota* (9%), and other minor phyla, including *Bacillota* (2%), *Verrucomicrobiota* (2%), and *Planctomycetota* (1%), which collectively accounted for a small fraction of the total diversity. Regarding novelty, a significant portion of the unique isolates represented potentially novel species. Specifically, 29% of the 92 unique bacterial strains exhibited less than 98% sequence identity to unknown species, suggesting that these 27 strains are suspected as novel species (Supplementary Table 4).

### 3.6 Identification of novel bacterial strains

**FIGURE 3:**
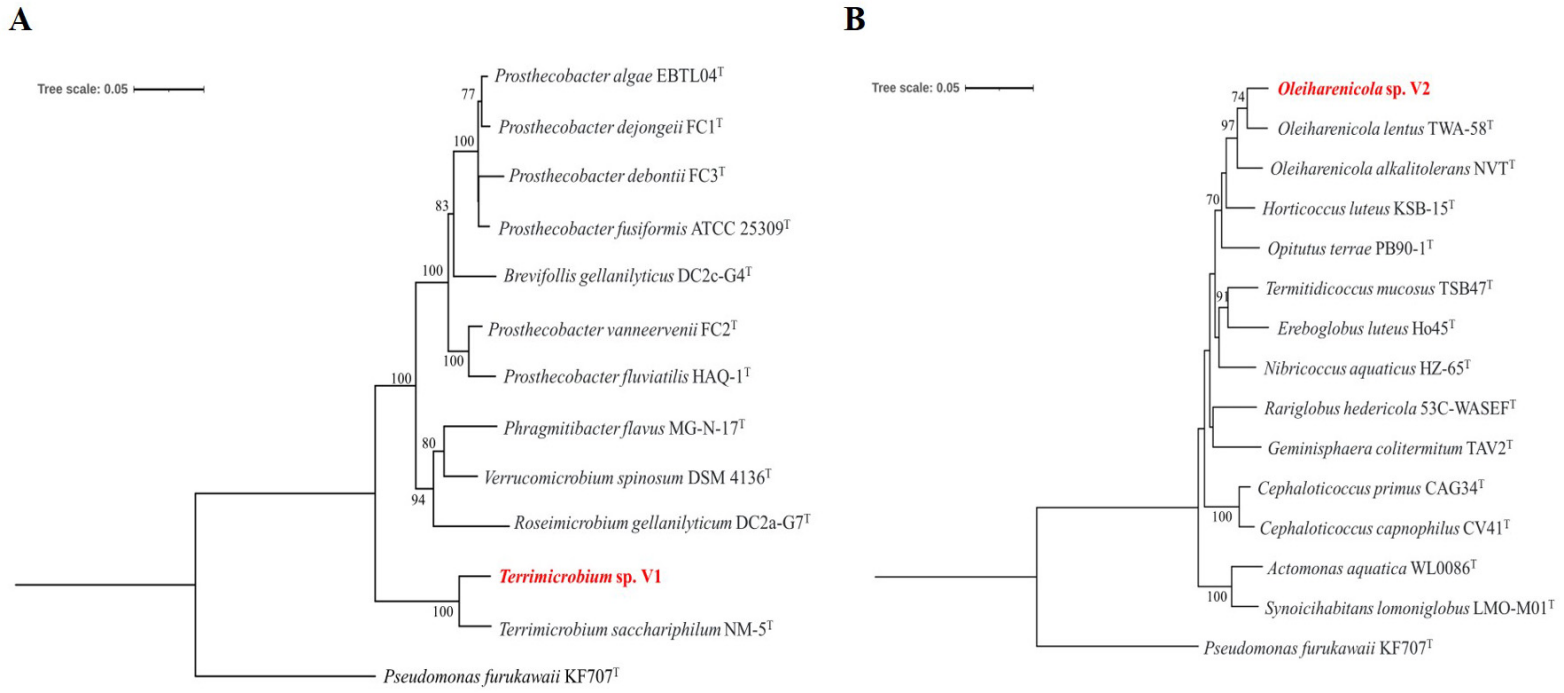
Phylogenetic tree constructed using 16S rRNA gene sequences by the Neighbor-Joining method for *Terrimicrobium* sp. V1 **(A)** and *Oleiharenicola* sp. V2 **(B)**. The percentage of replicate trees in which the associated taxa clustered together in the bootstrap test (1,000 replicates) are shown next to the branches. The tree is drawn to scale, with branch lengths in the same units as those of the evolutionary distances used to infer the phylogenetic tree. The evolutionary distances were computed using the Maximum Composite Likelihood method and are in the units of the number of base substitutions per site. This analysis involved 21 nucleotide sequences. All ambiguous positions were removed for each sequence pair (pairwise deletion option).

A total of 27 bacterial strains were identified and suspected as novel species based on 16S rRNA gene sequencing with a similarity of less than 98%. They belong to six different phyla, such as *Acidobacteriota*, *Actinomycetota*, *Bacteroidota*, *Planctomycetota*, *Pseudomonadota*, and *Verrucomicrobiota* (Supplementary Table 4). Among 27 novel bacterial strains, two *Verrucomicrobiota* strains were identified as *Terrimicrobium* and *Oleiharenicola*. According to the EzBioCloud database, the strains V1 and V2 have close similarity to *Terrimicrobium sacchariphilum* NM-5^T^ (95.83%) and *Oleiharenicola lentus* TWA-58^T^ (97.1%). These values fall below the 98.65% threshold typically required to propose a new species. Additionally, a phylogenetic tree also showed that the strains V1 and V2 are closely related to *Terrimicrobium sacchariphilum* NM-5^T^ and *Oleiharenicola lentus* TWA-58^T^, respectively (Figures 3A, B). Based on these initial confirmations, the strains V1 and V2 were suspected as novel species within the genus *Terrimicrobium* and *Oleiharenicola*, respectively.

### 3.7 Plant growth promotion and biocontrol potential of *Verrucomicrobiota* strains

**FIGURE 4:**
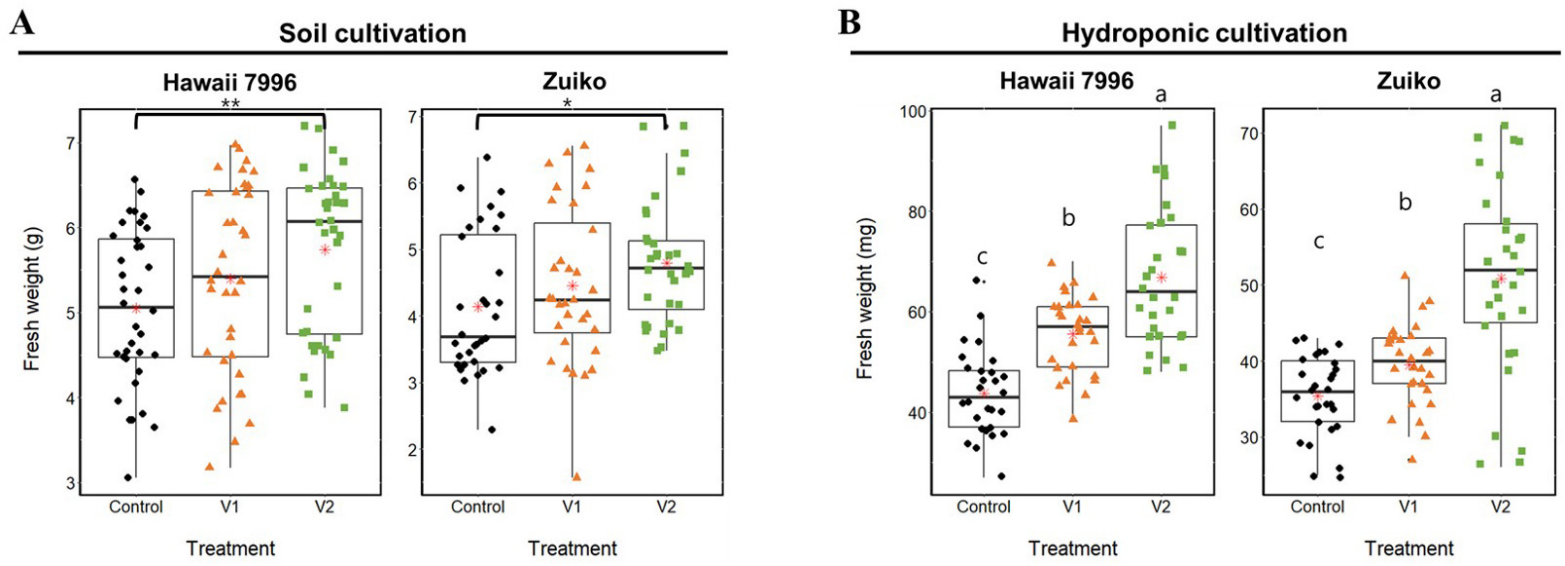
*In-planta* tomato plant growth promotion by treatment of *Verrucomicrobiota* strains V1 and V2. **(A)** Fresh weight of tomato plant in pot culture with nursery soil. The experiment was performed in triplicate with 10 plants per treatment (*n* = 30). Significant difference was noticed by analysis of variance (ANOVA; F_2,93_ = 3.133, *P* = 0.0482) followed by Tukey’s honestly significant difference (HSD) *post-hoc* test (*P < 0.05) for fresh weight of Zuiko at soil cultivation. For fresh weight of Hawaii 7996 at soil cultivation, the significant difference was noticed by Kruskal-Wallis rank sum test (*P* = 0.009774) with Dunn’s *post-hoc* test (**P < 0.01). **(B)** Fresh weight in hydroponic system. The experiment was performed in triplicate with 10 plants per treatment (*n* = 30). Significant difference was noticed by Welch’s ANOVA (F_2_ = 32.179, *P* = 8.063e^– 10^ for fresh weight of Hawaii 7996 and F_2_ = 18.652, *P* = 7.728e^– 07^ for fresh weight of Zuiko) followed by Games-Howell *post-hoc* test. Red color star marks and bolded lines of the boxplot are the average and median of indicated values, respectively.

In soil cultivation, strain V2 significantly increased the fresh weight of tomato seedlings compared to both the control and V1 treatments in Hawaii 7996 (mean ± SD: 5.73 ± 0.95 g for V2; 5.39 ± 1.12 g for V1; 5.04 ± 0.92 g for control; one-way ANOVA, F2,93 = 3.13, *P* = 0.048) and Zuiko (4.79 ± 0.91 g for V2; 4.45 ± 1.18 g for V1; 4.13 ± 1.05 g for control; Kruskal-Wallis, *P* = 0.0097; *n* = 30 per group) cultivars (Figure 4A). Specifically, V2-treated seedlings in both Hawaii 7996 and Zuiko cultivars exhibited the highest fresh weight, with a median value significantly greater than the control. In hydroponic cultivation, the fresh weight of tomato seedlings treated with V2 was significantly higher than both control and V1 treatments in Hawaii 7996 (mean ± SD: 66.82 ± 13.4 mg for V2, 55.53 ± 7.74 mg for V1, and 43.78 ± 8.51 mg for control, respectively; Welch’s ANOVA, F2 = 32.18, *P* < 0.001) and Zuiko (50.86 ± 12.88 mg for V2, 39.48 ± 5.44 mg for V1, and 35.37 ± 5.27 mg for control, respectively; Welch’s ANOVA, F_2_ = 18.65, *P* < 0.001; *n* = 30 per group) (Figure 4B). Strain V1 also contributed to an increase in fresh weight compared to the control, but the effect was less noticeable than V2. Overall, strain V2 consistently demonstrated the highest growth-promoting effect on fresh weight across both cultivation methods and cultivars (Figure 4).

**FIGURE 5:**
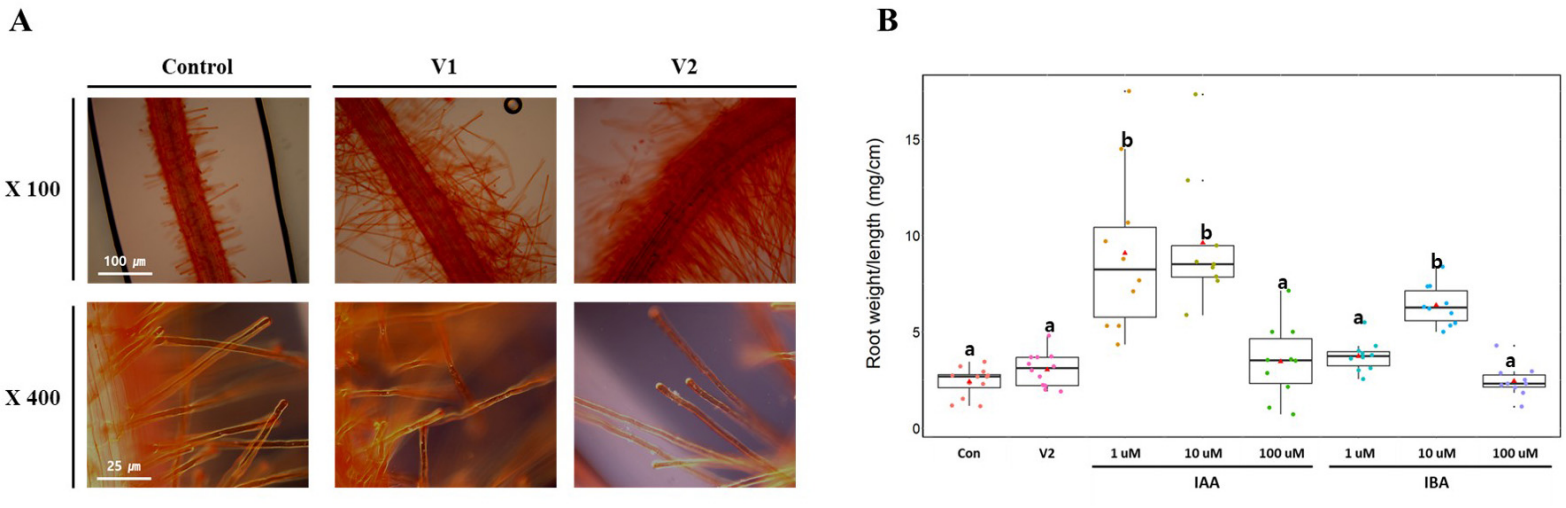
Light micrograph of tomato root growth and root biomass. **(A)** Morphology of root hairs in tomato plant by the treatment of V1 and V2. Seven-day-old tomato seedling roots were stained with Safranin-O for 5 min: where control (without bacteria) with very few and short root hairs; V1 and V2 are inoculated with different *Verrucomicrobiota* strains were confirmed to be thick and longer root hairs. **(B)** Root weight/ length comparison by the treatment of V2, IAA and IBA in hydroponic cultivation system. Different letters (a and b) indicate significant differences between samples, as determined by a Kruskal-Wallis rank sum test (*P* < 2.2e^– 16^), followed by Dunn’s *post-hoc* test.

The effect of bacterial strains V1 and V2 on root hair development in tomato plants was assessed through microscopic analysis (Figure 5A). The root hair morphology was significantly altered by the treatments, with both V1 and V2 strains promoting considerable enhancement in root hair growth compared to the untreated control. In the control group, root hairs were sparse and short, indicating limited development. However, plants treated with the strain V1 exhibited a noticeable increase in root hair density and length, as observed under 100 × and 400 × magnification. This was even more pronounced in plants treated with strain V2, where root hairs were visibly denser and longer than those in both the control and V1-treated plants. The significant increase in root hair density and length in response to these treatments suggests that strains V1 and V2 could play a vital role in enhancing nutrient uptake and overall root system architecture in tomato plants, potentially leading to improved plant growth.

In the hydroponic root-biomass assay, only strain V2 was tested and resulted in a significant increase in root weight per length (2.95 ± 0.75 mg cm^–1^) compared to the control (2.54 ± 0.78 mg cm^–1^ Kruskal-Wallis, *P* < 0.01; *n* = 10) (Figure 5B). The pattern of enhanced root mass and density observed in V2-treated plants was comparable to that produced by auxin treatments (IAA 10 μM, 8.70 ± 2.94 mg cm^–1^; IBA 10 μM, 6.06 ± 1.08 mg cm^–1^). These results suggest a phenotypic similarity but do not confirm a shared biochemical mechanism. Further metabolomic or genomic analyses would be required to determine whether V2 modulates auxin-related pathways.

**FIGURE 6:**
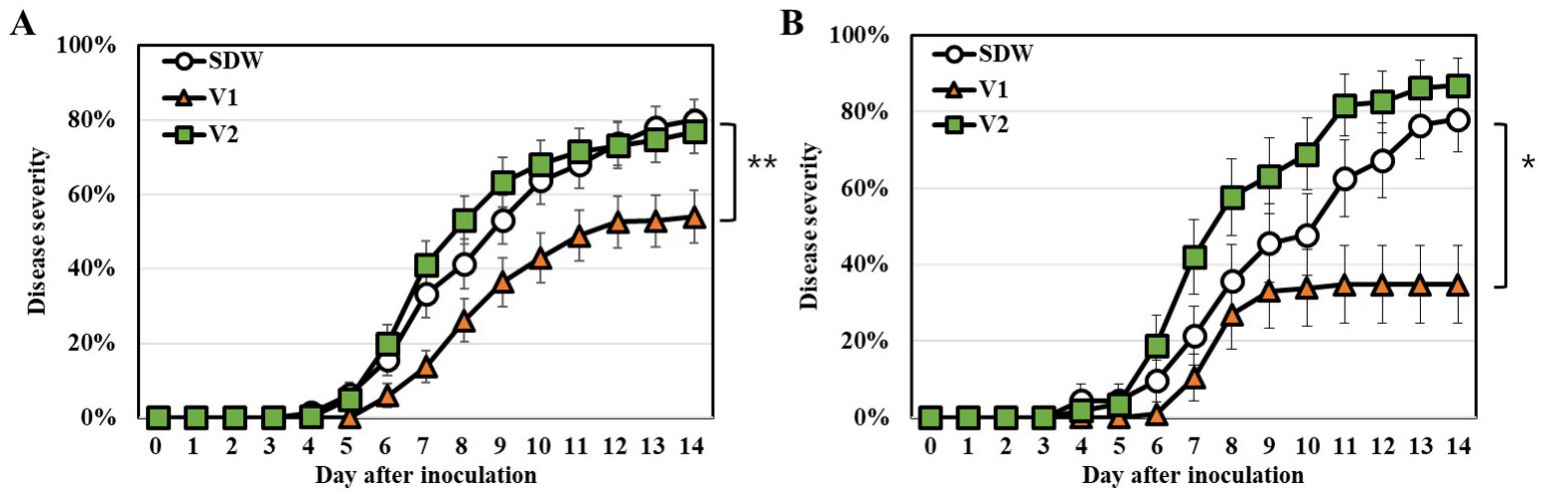
Bacterial wilt disease progress in the tomato plants treated with *Verrucomicrobiota* strains V1 and V2. **(A)** Disease severity against *R. pseudosolanacearum* GMI1000 on tomato cultivar Zuiko. **(B)** Disease severity against *R. solanacearum* SL341 on tomato cultivar Hawaii 7996. Repeated measures ANOVA showed a significant difference between control and *Verrucomicrobiota* strain V1 in Zuiko (**P < 0.01) and Hawaii 7996 (*P < 0.05). Each vertical bar represents the standard error of the means from three replicates with 10 plants per treatment (*n* = 30).

The impact of bacterial strains V1 and V2 on disease severity in tomato cultivars Zuiko and Hawaii 7996 was assessed following inoculation with *R. pseudosolanacearum* GMI1000 and SL341 strains, respectively (Figure 6). Treatment with strain V1 significantly reduced disease severity compared to the control in both Zuiko (Figure 6A) and Hawaii 7996 (Figure 6B) cultivars (mean ± SD: 35.4 ± 4.2% vs. 78.7 ± 5.1% for Zuiko; 54.9 ± 3.7% vs. 80.2 ± 4.5% for Hawaii 7996; repeated-measures ANOVA, F = 4.60, *P* = 0.043 and F = 8.35, *P* = 0.0057, respectively; *n* = 30). Strain V2 slightly reduced the disease severity compared to the control in Hawaii 7996 but failed to control the bacterial wilt in Zuiko. Taken together, two strains of *Verrucomicrobiota* showed differential activity on tomato for plant growth and BW suppression.

## 4 Discussion

In our previous study, a putative key taxon associated with regulating tomato BW resistance was identified in the rhizosphere of Hawaii 7996 treated with a specific upland soil MF (Choi et al., 2020). Many microorganisms play crucial roles in plant-microbe interactions within microbial communities (Dessaux et al., 2016; Kwak et al., 2018). However, since many of these microorganisms cannot be cultured, studying their interactions with soil and plants is challenging. Thus, obtaining a diverse range of microorganisms from plant-associated microbial communities is essential for understanding these interactions (Kwak et al., 2018; Lee et al., 2024). Efforts to improve novel bacterial recovery from natural environments include modifying growth conditions or cultured media (Hamaki et al., 2005; Kato et al., 2018) and using dilution or physical separation techniques (dos Santos Furtado and Casper, 2000; Heidarrezaei et al., 2020), but these methods still fall short in entirely capturing unknown microorganisms.

In this study, to isolate the bacterial strains of *Verrucomicrobiota* phylum, which was among the observed key taxa in our previous study, tomato rhizosphere microbiome fraction was enriched with various carbon sources (Yang Z. et al., 2023; Han et al., 2023). This approach aimed to isolate the specific microbial taxa contributing to the enhanced plant growth and disease resistance observed in the tomato. Similarly, adding antibiotics led to a more diverse microbial community, with a broader distribution across different phyla, particularly in the secondary enrichment samples. This approach indicates that streptomycin pressure might alter microbial diversity in soil by reducing the dominant specific phyla like *Pseudomonadota* and enabling less dominant phyla to be prevalent (Arefa et al., 2021; Chen et al., 2023). In fact, the presence of streptomycin reduced the abundance of phylum *Pseudomonadota* and increased the other phylum, particularly in the samples with complex carbon sources lactose, alginate, pectin, dulcitol, glycerol, and casein-enriched samples (Figure 1C). It could be attributed to the selection pressure exerted by antibiotics, which may reduce competition and allow for the survival and growth of a broader range of microbial taxa. The microbial community composition of tomato field rhizosphere soil is heavily influenced by the type of carbon source provided and the presence or absence of antibiotics. These findings suggest that carbon and antibiotic selection are critical factors in shaping soil microbial community. In this study, we isolated 27 novel microbial strains including two distinct strains of *Verrucomicrobiota*, such as *Terrimicrobium* sp. V1 and *Oleiharenicola* sp. V2. This finding is correlated with a previous report (Bunger et al., 2020), which identified four novel *Verrucomicrobiota* strains isolated from rice plants.

**FIGURE 7:**
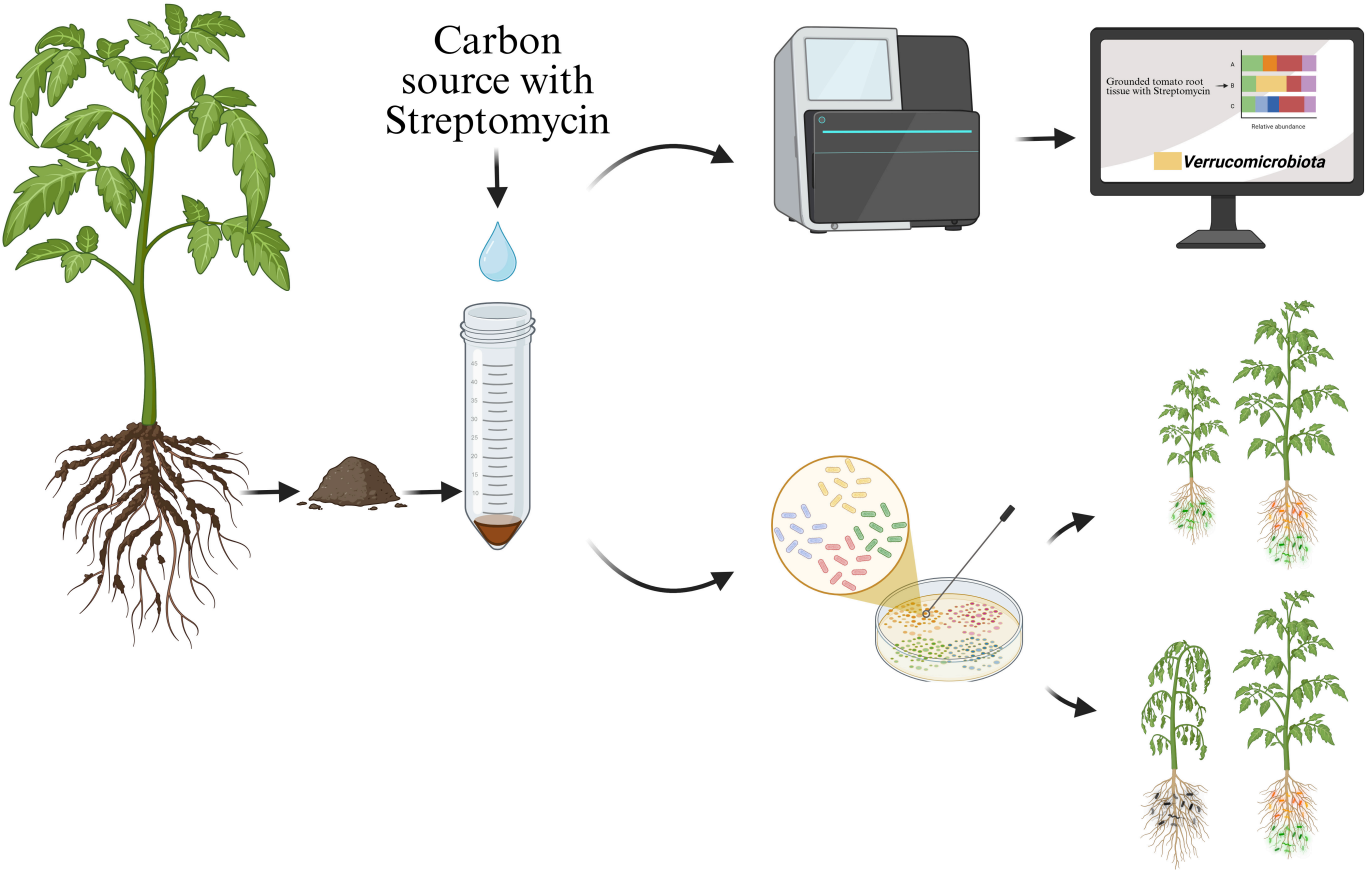
Schematic diagram showing the experimental workflow of the present study.

Moreover, it should be noted that the isolation of slow-growing bacteria such as *Verrucomicrobiota*, *Bacteroidota*, and *Actinomycetota* from plant or soil environments are challenging to grow on a general culture media (Janssen et al., 2002; O’Sullivan et al., 2004; Hernandez-Pacheco et al., 2021). This is because *Pseudomonadota* particularly, *Pseudomonas* predominated in these environments, representing more than 50% of the total isolates (Khan Chowdhury et al., 2017). To the best of our knowledge, this study is among the first to demonstrate the use of combined carbon and antibiotic enrichment approaches, guided by microbiome analysis, to successfully isolate novel *Verrucomicrobiota* taxa from field MF-treated tomato plants (Figure 7). In contrast, other studies also conducted 16S rRNA gene amplicon sequencing on tomato (Hernandez-Pacheco et al., 2021) and maize (Aguirre-von-Wobeser et al., 2018), have detected *Verrucomicrobiota* but were unsuccessful to isolate this group of bacteria in laboratory culture media.

This study highlights the critical role of tomato root tissue as an effective carbon source for the isolation of *Verrucomicrobiota*, facilitating the enrichment and successful cultivation of these novel microorganisms (Figure 7). Root-derived carbon source supports the growth of *Verrucomicrobiota* strains and underscores their ecological role in the rhizosphere, where they interact beneficially with plant roots (Tanaka et al., 2024). These associations may contribute to improved root surface development and nutrient uptake, as suggested by prior studies reporting similar effects of beneficial rhizosphere bacteria (Bunger et al., 2020). The ability of *Verrucomicrobiota* to thrive on complex carbohydrates derived from root tissues points to their potential as key players in plant-microbe symbiosis, promoting plant growth and resilience in agricultural settings.

Biocontrol strains are beneficial for promoting plant growth and managing BW. Introducing the exogenous biocontrol bacteria to the soil can influence the soil microbiota (Chandrasekaran et al., 2016; Yang et al., 2022). For instance, the addition of *Bacillus cereus* AR156 led to notable differences in microbial community composition and abundance in the rhizosphere between treatment and control samples (Yang B. et al., 2023). In this study, biocontrol strains *Terrimicrobium* sp. V1 and *Oleiharenicola* sp. V2 significantly promoted tomato growth and provided adequate BW protection in both Hawaii 7996 and Zuiko cultivars. Our findings align with other studies that have shown inoculation with *Bacillus velezensis* B63 or *Pseudomonas fluorescens* P142 in tomato root soil can significantly alter rhizosphere bacterial community composition, with microbiome shifts potentially triggering plant defense against *R. solanacearum* B3B (Elsayed et al., 2019). The interactions among *Verrucomicrobiota* biocontrol strains, the natural soil microbiome, and tomato roots are complex, and the precise mechanisms by which biocontrol strains modulate soil microbiota to promote plant growth and resist soilborne diseases remain unclear. Moreover, the observed enhancement in root development and disease resistance by *Verrucomicrobiota* strains may involve auxin-like phytohormone production, competition with pathogens for niche and nutrients, and induction of plant systemic resistance. These complementary mechanisms likely contribute to the dual role of *Terrimicrobium* sp. V1 and *Oleiharenicola* sp. V2 in promoting tomato growth and mitigating bacterial wilt disease. Further studies on the impact of biocontrol strains such as *Terrimicrobium* sp. V1 and *Oleiharenicola* sp. V2 on the tomato rhizosphere microbiota in natural soil condition would, therefore, be highly valuable.

While this study successfully demonstrates the isolation and characterization of novel *Verrucomicrobiota* strains through carbon and antibiotic guided enrichment, several limitations should be noted. First, the microbial community analyses were based on 16S rRNA gene sequencing, which, although informative for taxonomic profiling, provides limited functional or strain-level resolution. Second, we did not perform whole-genome sequencing or metabolite profiling, which would be necessary to validate the genetic basis and biochemical mechanisms underlying the observed plant growth promotion and disease suppression. Third, as with most enrichment-based cultivation approaches, potential biases toward faster-growing or more adaptable taxa may have influenced the apparent community composition. Future multi-omics and comparative cultivation studies will be valuable to address these limitations and further elucidate the ecological and functional diversity of *Verrucomicrobiota* in the rhizosphere.

## Data Availability

The original contributions presented in the study are publicly available. The 16S rRNA gene amplicon sequences have been deposited in the NCBI Sequence Read Archive (SRA) under the accession number PRJNA1330418.

## Footnotes

1
https://www.ezbiocloud.net/identify
