# Supplementary material for: Novel Verrucomicrobiota strains associated with plant root tissue enhance plant growth and suppress bacterial wilt in tomato
Source: Front Microbiol. 2025 Dec 17;16:1712154. doi: 10.3389/fmicb.2025.1712154 (PMC12753962; doi:10.3389/fmicb.2025.1712154)
Supplement: Supplementary file 1 [file Table_1.docx]

**Novel *Verrucomicrobiota* strains associated with plant root tissue enhances plant growth and suppresses bacterial wilt in tomato**

**Minseo Choi^1,^†, Manigundan Kaari^2.3,^†, Hyoung Ju Lee^2^ and Seon-Woo Lee^1,2^***

^1^ Department of Applied Bioscience, Dong-A University, Busan, Republic of Korea

^2^ Institute of Agricultural Life Sciences, Dong-A University, Busan, Republic of Korea

^3^ Centre for Drug Discovery and Development, Sathyabama Institute of Science and Technology, Chennai, Tamilnadu, India

†These authors contributed equally to this work.

***Correspondence:**

Seon-Woo Lee, Ph.D

Professor

Department of Applied Bioscience

Dong-A University

Busan 49315

Republic of Korea

Email: [seonlee@dau.ac.kr](mailto:seonlee@dau.ac.kr)

TEL: +82-51-200-7551

FAX: +82-51-200-7505

Supplementary Figure S1. Schematic overview of analysis system for plant-microbiome interaction (ASPMI) (Choi et al., 2020) and preparation of rhizosphere microbial fractions for enrichment culture.

**
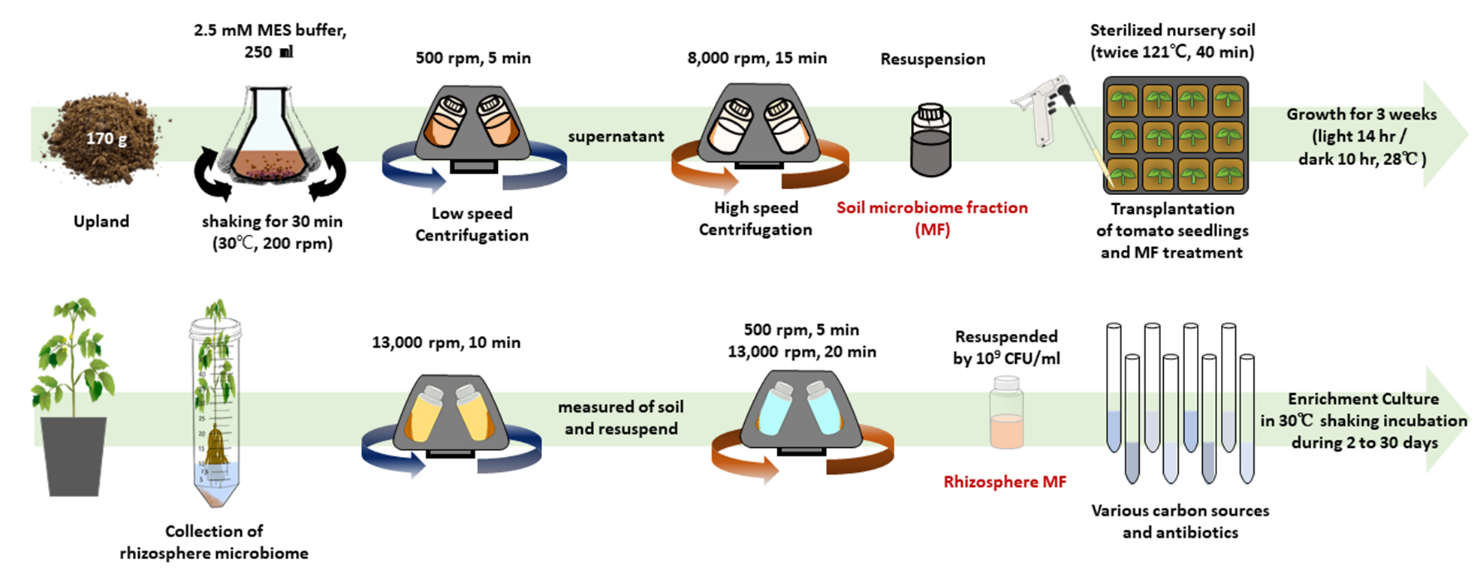
**

Supplementary Figure S2. PERMANOVA analysis of permutational multivariate of group dispersions based on Bray-Curtis dissimilarity using pairwiseAdonis2 in R package with samples grouped by carbon source enriched (A) and streptomycin treated (B) primary cultures. Mono, monosaccharide; Di, disaccharide; Oligo, oligosaccharide; Poly, polysaccharide; Polyol, polyolsaccharide; Comb., combination of the carbon sources; PRT, plant root tissue; Con, control; N, without streptomycin; A, with steptomycin. *, *p*<0.05; **, *p* <0.01; ***, *p* <0.001.

**
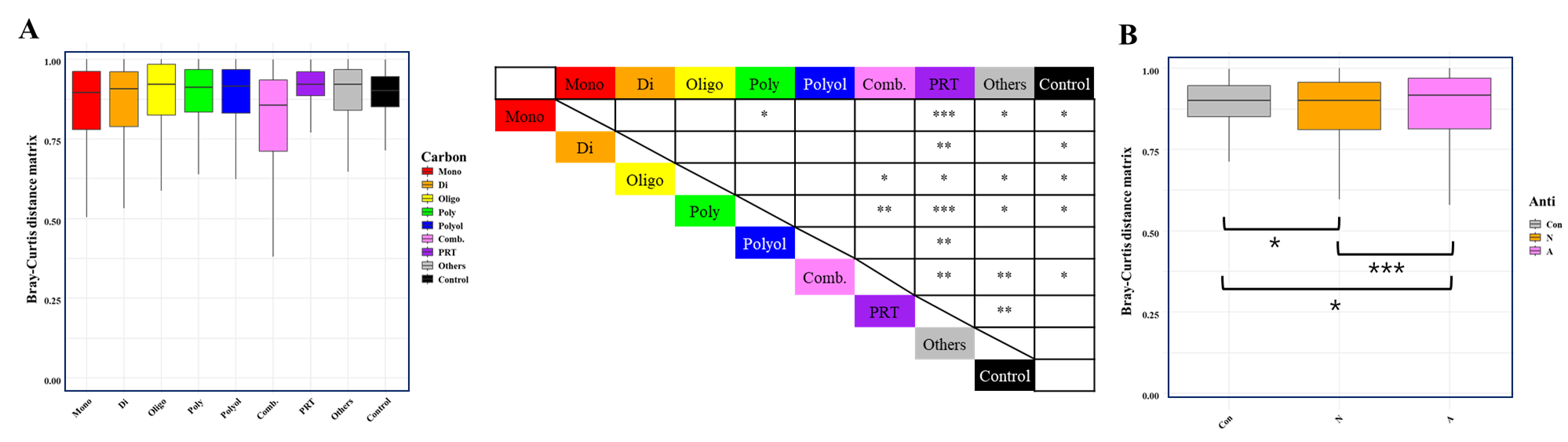
**

Supplementary Figure S3. PERMANOVA analysis of permutational multivariate of group dispersions based on Bray-Curtis dissimilarity using pairwiseAdonis2 in R package with samples grouped by carbon source enriched (A) and streptomycin treated (B) secondary cultures. Mono, monosaccharide; Di, disaccharide; Oligo, oligosaccharide; Poly, polysaccharide; Polyol, polyolsaccharide; Comb., combination of the carbon sources; PRT, plant root tissue; Con, control; N, without streptomycin; A, with streptomycin. *, *p* <0.05; **, *p* <0.01; ***, *p* <0.001.


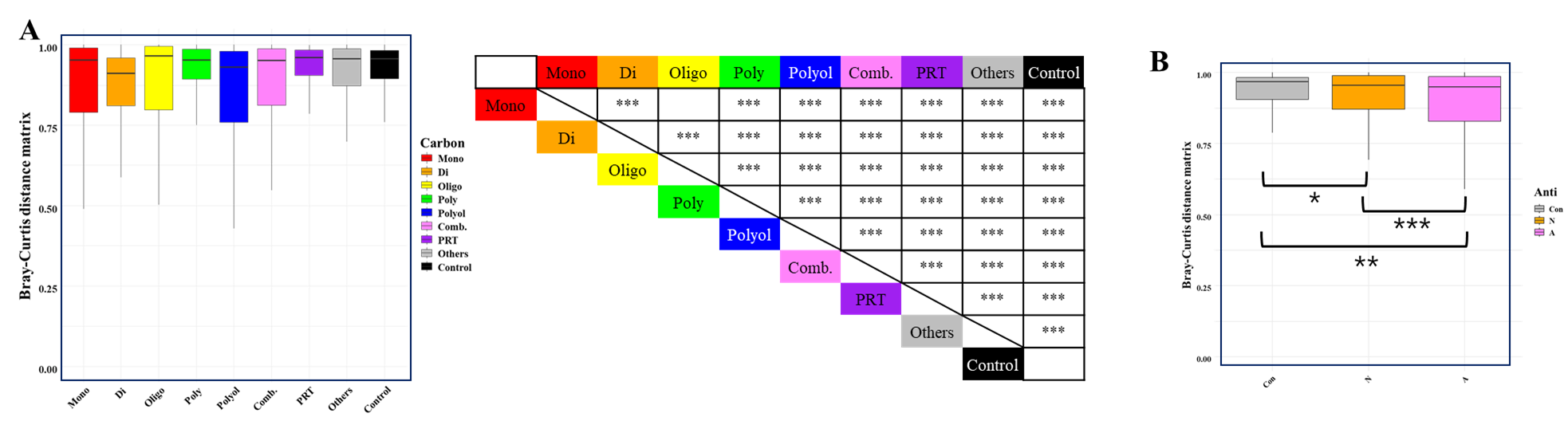


**Reference**

Choi, K., Choi, J., Lee, P.A., Roy, N., Khan, R., Lee, H.J., Weon, H.Y., Kong, H.G., Lee, S-W. (2020). Alteration of bacterial wilt resistance in tomato plant by microbiota transplant. *Front. Plant Sci*. 11, 1186. <https://doi.org/10.3389/fpls.2020.01186>.
